# Biota from the coastal wetlands of Praia da Vitória (Terceira, Azores, Portugal): Part 3 – Birds

**DOI:** 10.3897/BDJ.7.e34327

**Published:** 2019-05-28

**Authors:** Sofia Goulart, João Pedro Barreiros, Mariana R. Brito, Sónia Santos, César M.M. Pimentel, Elisabete Nogueira, Paulo Alexandre Vieira Borges

**Affiliations:** 1 LIFE CWR – LIFE project “Ecological Restoration and Conservation of Praia da Vitória Coastal Wet Green Infrastructures, Praia da Vitória, Azores, Portugal LIFE CWR – LIFE project “Ecological Restoration and Conservation of Praia da Vitória Coastal Wet Green Infrastructures Praia da Vitória, Azores Portugal; 2 CE3C – Centre for Ecology, Evolution and Environmental Changes / Azorean Biodiversity Group and Universidade dos Açores, Faculdade de Ciências Agrárias e do Ambiente, Angra do Heroísmo, Azores, Portugal CE3C – Centre for Ecology, Evolution and Environmental Changes / Azorean Biodiversity Group and Universidade dos Açores, Faculdade de Ciências Agrárias e do Ambiente Angra do Heroísmo, Azores Portugal

**Keywords:** Aves, Azores, Terceira, Wetlands, Ornitofauna.

## Abstract

**Background:**

The data presented here come from field observations of Aves between August 2013 and October 2018 as part of a LIFE research project aiming to preserve and restore three coastal wetlands from Praia da Vitória (Terceira Island, Azores, Portugal). Systematic monthly observations were carried out for five years in order to provide a checklist and monitoring of bird species and subspecies observed in three sites: Paul da Praia da Vitória (PPV), Paul do Belo Jardim (PBJ) and Paul da Pedreira do Cabo da Praia (PPCP). Main objectives were to determine their ornithological richness while also adding data to the overall knowledge of Azorean Avifauna and to monitor seasonal and between-year variation on species abundance.

**New information:**

During a five-year observation period (2013-2018), a total of 82,985 birds belonging to 108 species/subspecies were observed. From this, 16,663 were in PPV, 11,793 from PBJ and 54,529 from PPCP. The total richness was 55, 40 and 85, respectively. Three species are first records for the Azores: *Aythya americana* (Eyton, 1838); *Chlidonias leucopterus* (Temminck, 1815) and *Tringa brevipes* (Vieillot, 1816). One species is a new record for Terceira Island: *Lophodytes cucullatus* (Linnaeus, 1758).

## Introduction

The Azorean Avifauna has been described in several publications, the most recent being Rodrigues et al. (2010), Rodebrand (2012) and Barcelos et al. (2015). Despite the fact that the Azorean list of breeding birds is short (37 species breeding and seven occasionally nesting; Rodrigues et al. 2010), as a consequence of dramatic extinction events (Rando et al. 2013, Alcover et al. 2015, Rando et al. 2017), those publications added numerous records of non-breeding landbird and waterbird species, particularly occasional migrant and wintering species. Those novelties are a consequence of an increase in birdwatching activity on several Azorean islands and an increased interest in rare Nearctic birds arriving to Azores (Alfrey et al. 2018), due to storms that divert birds from their normal migratory routes.

Three coastal wetlands from the municipality of Praia da Vitória (Terceira Island, Azores, Portugal) - *Paul da Praia da Vitória* (PPV), *Paul do Belo Jardim* (PBJ) and *Paul da Pedreira do Cabo da Praia* (PPCP) – were studied during the LIFE – Coastal Wetlands Restoration Project and are known as a high avifauna site attracting birdwatchers and which include an important number of species (Dias et al. 1991, Morton et al. 1997, Morton et al. 1998, Melo and Dias 2005, Pereira and Melo 2017). Pereira and Melo (2017) published a field guidebook on the bird species occurring in PPCP, highlighting the particular importance of this wetland for migrant species and regular and occasional wintering birds.

This manuscript is the third contribution in a series of papers that characterise the biota of the three coastal wetlands from this area (Borges et al. 2018, Gabriel et al. 2019).

## General description

### Purpose

**Figure 1.**
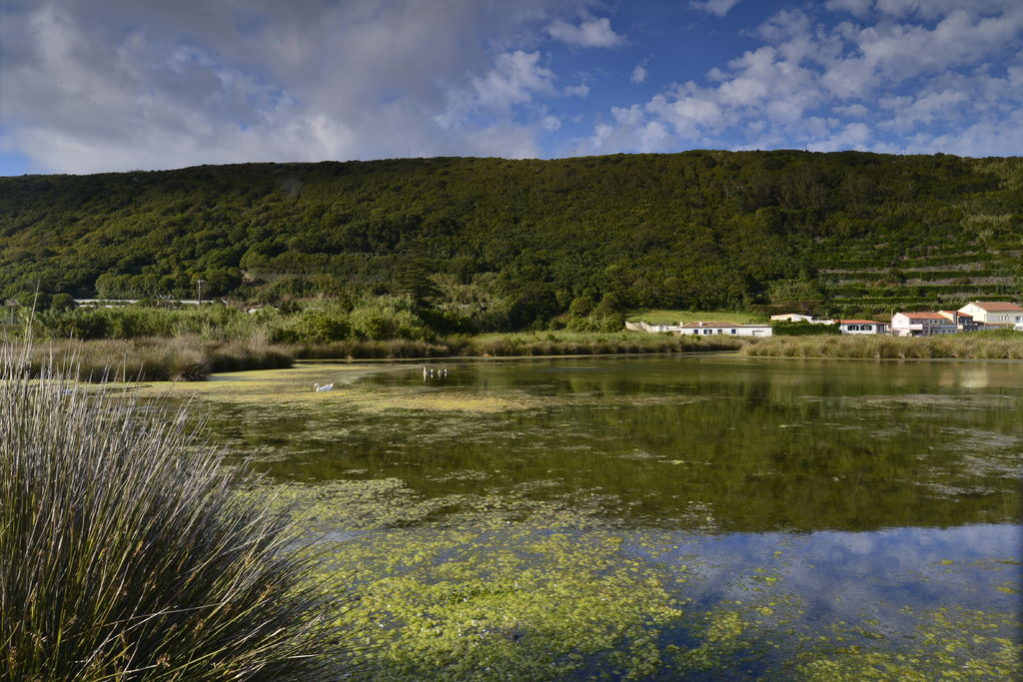
General aspect of Paul da Praia da Vitória (Photo by Paulo A.V. Borges).

**Figure 2.**
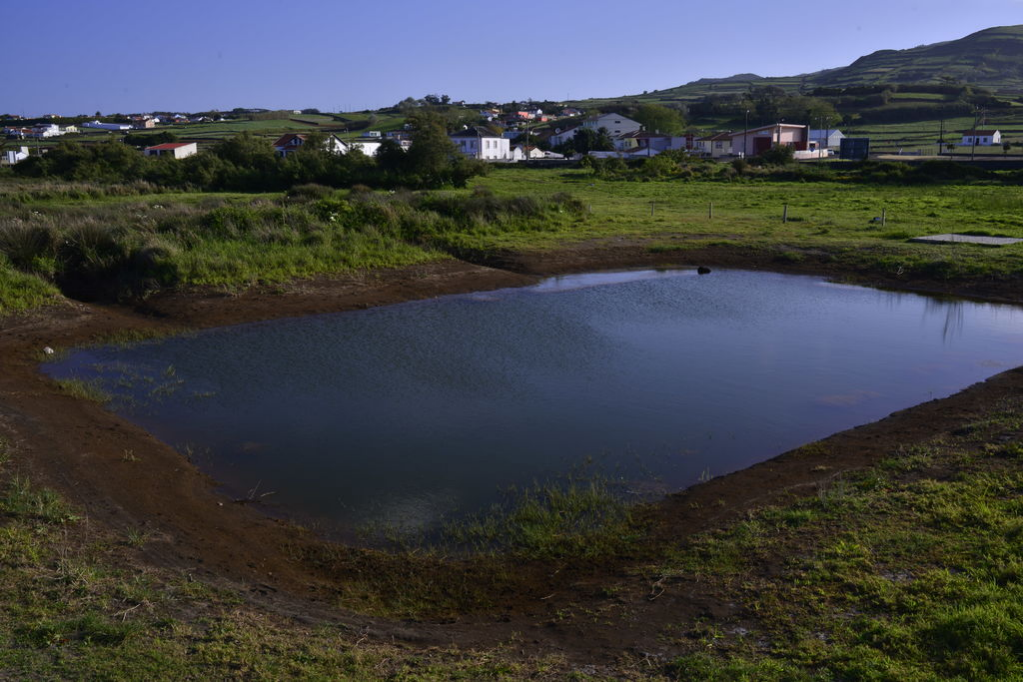
General aspect of Paul Belo Jardim (Photo by Paulo A.V. Borges).

**Figure 3.**
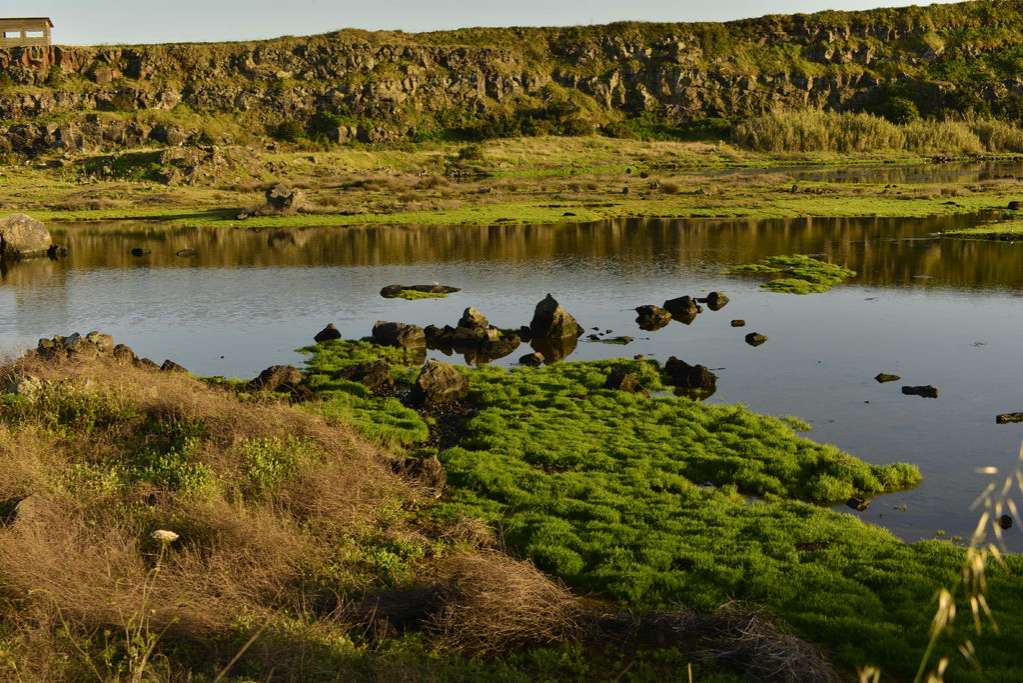
General aspect of Paul da Pedreira do Cabo da Praia (PPCP) (Photo by Paulo A.V. Borges).

The aim of this work is to inventory the avifauna present in the three coastal areas of Praia da Vitória (Terceira Island, Azores), focused on the LIFE-CWR Project, Paul da Praia da Vitória (PPV) (Fig. 1), Paul do Belo Jardim (PBJ) (Fig. 2) and Paul da Pedreira do Cabo da Praia (PPCP) (Fig. 3), in order to improve our knowledge on the bird diversity that occurs in this area, detect eventual new species for the Azores and monitor seasonal and between-year variation on species abundance.

## Project description

### Title

Inventory of bird species in three coastal wetlands from Terceira Island (Azores)

### Personnel

The inventory was conducted during five years between August 2013 and October 2018 by experienced birdwatchers: Sofia Goulart, Mariana R. Brito and Sónia Santos.

### Study area description

Terceira Island (area: 400.6 km²; elevation: 1,021.14 m) is one of the nine islands of the Azores archipelago, located in the North Atlantic, roughly at 38°43'49''N 27°19'10''W (Forjaz et al. 2004). The climate in the Azores is temperate oceanic, with regular and abundant rainfall, high levels of relative humidity and persistent western winds, mainly during the winter and autumn seasons (Azevedo et al. 1999).

## Sampling methods

### Study extent

This study covers a small coastal area with 3.58 km extension between PPV and PPCP.

### Sampling description

At the three wetland sites, more than 788 days of observations were carried out for a total of ca. 11,820 h of direct observations. Each observation lasted 15 minutes in which every sighted bird was registered. These were made by experienced birdwatchers (two to three researchers in the field each day) using a Swarovski 20-60 telescope and Opticron Verano HD 10-42 binocular. Photographs were made with a Canon 60D camera (a database and Photo repository is available at http://lifecwr.com/index.php/pt/observacao/registos-de-observacao/registos-de-observacao-2). Whenever needed, several field-guides were used (e.g. Pereira 2010, Mullarney et al. 2012), as well as websites on Azorean birds, namely AVES DOS AÇORES, Azores bird sightings and Birding Azores.

## Geographic coverage

### Description

Praia da Vitória marshes, Terceira Island (Azores), Macaronesia, Portugal.

### Coordinates

38º42'09''N and 38°44'12''N Latitude; 27º03'46''W and 27°02'39''W Longitude.

## Taxonomic coverage

### Description

Aves

## Temporal coverage

### Notes

Data range: August 2013 – October 2018.

## Usage rights

### Use license

Creative Commons Public Domain Waiver (CC-Zero)

## Data resources

### Data package title

LIFE_CWR_TER_Aves

### Resource link

http://ipt.gbif.pt/ipt/resource?r=azores_birds

### Alternative identifiers

http://islandlab.uac.pt/software/ver.php?id=34

### Number of data sets

1

### Data set 1.

#### Data set name

Birds from Praia da Vitória marshes (Terceira, Azores, Portugal)

#### Data format

Darwin Core Archive

#### Number of columns

50

#### Download URL

http://ipt.gbif.pt/ipt/resource?r=azores_birds

#### Data format version

version 1

#### Description

In this data table, we include all the records for which a taxonomic identification of the species was possible. The dataset submitted to GBIF is structured as a sample event dataset, with two tables: event (as core) and occurrences. The data in this sampling event resource have been published as a Darwin Core Archive (DwCA), which is a standardised format for sharing biodiversity data as a set of one or more data tables. The core data table contains 2003 records. One extension data table also exists. An extension record supplies extra information about a core record. The number of records in each extension data table is illustrated in the IPT link. This IPT archives the data and thus serves as the data repository. The data and resource metadata are available for downloading in the downloads section. The versions table lists other versions of the resource that have been made publicly available and allows tracking changes made to the resource over time. In Suppl. material 1, we provide a simpler dataset with few columns in a single table.

**Data set 1.** 

Column label	Column description
*Table Event*	Table Event
id	Unique identifier
type	Type of the record, as defined by the Public Core standard
licence	Reference to the licence under which the record is published
InstitutionID	The identity of the institution publishing the data
InstitutionCode	The code of the institution publishing the data
datasetName	Name of the dataset
eventID	Identifier of the events, unique for the dataset
eventDate	Date or date range the record was collected
startDayOfYear	The earliest ordinal day of the year on which the Event occurred (1 for 1 January, 365 for 31 December, except in a leap year, in which case it is 366)
year	Year
month	Month
day	Day
islandGroup	Archipelago of the sampling site
island	Island of the sampling site
country	Country of the sampling site
countryCode	ISO code of the country of the sampling site
county	Name of the county
locality	Name of the locality
minimumElevationInMeters	Minimum elevation in metres
maximumElevationInMeters	Maximum elevation in metres
verbatimCoordinates	Original coordinates recorded
decimalLatitude	Approximate centre point decimal latitude of the field site in GPS coordinates
decimalLongitude	Approximate centre point decimal longitude of the field site in GPS coordinates
geodeticDatum	The reference point for the various coordinate systems used in mapping the earth
Table Occurrences	Table of Occurrences
id	Unique identifier
modified	Date of the last modification of the record
language	A language of the resource
basisOfRecord	The nature of the data record
occurrenceID	Identifier of the record, coded as a global unique identifier
catalogNumber	Record number of the specimen in the collection
recordedBy	Name of the person who performed the sampling of the specimens
individualCount	Total number of individuals captured
organismQuantity	Total number of individuals captured, as numeric
organismQuantityType	The unit of the identification of the organisms
eventID	Identifier of the events, unique for the dataset
identifiedBy	Name of the person who made the identification
dateIdentified	Date on which the record was identified
scientificName	Complete scientific name including author and year
kingdom	Kingdom name
phylum	Phylum name
class	Class name
order	Order name
family	Family name
genus	Genus name
specificEpithet	Specific epithet
infraspecificEpithet	Infraspecific epithet, when available
taxonRank	Lowest taxonomic rank of the record
scientificNameAuthorship	Name of the author of the lowest taxon rank included in the record

## Additional information

We observed and identified 82,985 birds belonging to 26 families, including 108 species or subspecies. Families Scolopacidae (32 species) and Anatidae (24 species) were the most diverse while three species corresponded to 47.8% of all observed/identified birds (Table 1): *Calidris alba* (18,856), *Charadrius alexandrinus alexandrinus* (10,726) and *Arenaria interpres* (10,074). The Order Charadriformes, with 63,671 individuals observed, corresponds to 75.7% of all birds from this work (Table 1). This abundance of waders is certainly an expected feature on wetlands. Eleven species were represented by a single individual observation and 38 by ten or less individuals.

Of all observed species, three are not referred to in Barcelos et al. (2015), which is the most recent update on the list of Azorean birds: *Aythya americana* (Eyton 1838), *Chlidonias leucopterus* (Temminck 1815) and *Tringa brevipes* (Vieillot 1816). One species is a new record for Terceira Island: *Lophodytes cucullatus* (Linnaeus 1758). *Aythya americana* is a Nearctic occasional migrant duck, *Chlidonias leucopterus* is a Palearctic tern and *Tringa brevipes* is a shorebird breeding in northeast Siberia. All other species have previously been recorded at several levels of relative abundance, both as breeding native (ten taxa), breeding Azorean endemic (seven taxa), breeding Macaronesian endemic (one taxon), breeding introduced (four taxa) and vagrant (87 additional taxa) (Table 1). About 15 out of the 21 breeding species are common with more than 100 individuals recorded in the three sites. Based on the Barcelos et al. (2015) classification of vagrant species, in the three sites we found: 45 occasional migrants, 21 regular migrants, 17 occasional wintering taxa and 26 regular wintering taxa. The Palearctic taxa dominate the community of birds (52 taxa), whereas Holarctic (28 taxa) and Nearctic (25 taxa) have similar but with half of the frequency.

## Supplementary Material

Supplementary material 1 LIFE_CWR_Birds Data type: Occurrences and abundances
Brief description: In this contribution, we present detailed data on the distribution and abundance of species belonging to several groups of arthropods in three Terceira island (Azores) wetlands: Paul da Praia da Vitória (PPV), Paul do Belo Jardim (PBJ) and Paul da Pedreira do Cabo da Praia (PPCP).
File: oo_268688.xlsx Goulart S et al.

## Figures and Tables

**Table 1.** List of Aves and their abundance in the three coastal wetlands of Praia da Vitória, Terceira Island, Azores, Portugal (*Paul da Praia da Vitória* (PPV), *Paul do Belo Jardim* (PBJ) and *Paul da Pedreira do Cabo da Praia* (PPCP), indicating Order, Family and both breeding status (b) and colonisation status (END – endemic from Azores; MAC – endemic from Macaronesia; n = native non-endemic; i = introduced).

**Order**	**Family**	**Taxon**	**Status**	**PPV**	**PBJ**	**PPCP**	**Total**
Anseriformes	Anatidae	*Aix galericulata*				1	1
Anseriformes	Anatidae	*Aix sponsa*		1			1
Anseriformes	Anatidae	*Anas acuta*		302	1	96	399
Anseriformes	Anatidae	*Anas americana*		1237		33	1270
Anseriformes	Anatidae	*Anas carolinensis*				9	9
Anseriformes	Anatidae	*Anas clypeata*		1			1
Anseriformes	Anatidae	*Anas crecca*		94		235	329
Anseriformes	Anatidae	*Anas discors*		117		147	264
Anseriformes	Anatidae	*Anas penelope*		689		30	719
Anseriformes	Anatidae	*Anas querquedula*		14		16	30
Anseriformes	Anatidae	*Anas strepera*		22			22
Anseriformes	Anatidae	*Anser cygnoides*		37			37
Anseriformes	Anatidae	*Aythya affinis*		295		1	296
Anseriformes	Anatidae	*Aythya americana*		27			27
Anseriformes	Anatidae	*Aythya collaris*		531	6	38	575
Anseriformes	Anatidae	*Aythya ferina*		12		3	15
Anseriformes	Anatidae	*Aythya fuligula*		654		7	661
Anseriformes	Anatidae	*Aythya marila*		296			296
Anseriformes	Anatidae	*Branta bernicla*		7		3	10
Anseriformes	Anatidae	*Branta canadensis*		8			8
Anseriformes	Anatidae	*Bucephala clangula*				5	5
Anseriformes	Anatidae	*Clangula hyemalis*		4			4
Anseriformes	Anatidae	*Lophodytes cucullatus*		64			64
Anseriformes	Anatidae	*Tadorna tadorna*				14	14
Charadriiformes	Charadriidae	*Charadrius alexandrinus alexandrinus*	b / n	7	529	10190	10726
Charadriiformes	Charadriidae	*Charadrius hiaticula*			9	964	973
Charadriiformes	Charadriidae	*Charadrius semipalmatus*			2	124	126
Charadriiformes	Charadriidae	*Pluvialis apricaria*				18	18
Charadriiformes	Charadriidae	*Pluvialis dominica*				14	14
Charadriiformes	Charadriidae	*Pluvialis fulva*				18	18
Charadriiformes	Charadriidae	*Pluvialis squatarola*			225	2970	3195
Charadriiformes	Charadriidae	*Vanellus vanellus*				3	3
Charadriiformes	Haematopodidae	*Haematopus ostralegus*				1	1
Charadriiformes	Laridae	*Chroicocephalus philadelphia*				1	1
Charadriiformes	Laridae	*Chroicocephalus ridibundus*		95	425	71	591
Charadriiformes	Laridae	*Larus argentatus*			2		2
Charadriiformes	Laridae	*Larus delawarensis*			12		12
Charadriiformes	Laridae	*Larus fuscus*			153		153
Charadriiformes	Laridae	*Larus glaucoides glaucoides*			13	1	14
Charadriiformes	Laridae	*Larus hyperboreus*			7	1	8
Charadriiformes	Laridae	*Larus marinus*			70	2	72
Charadriiformes	Laridae	*Larus michahellis atlantis*	b / END	171	8893	731	9795
Charadriiformes	Laridae	*Rissa tridactyla*		2	4	2	8
Charadriiformes	Scolopacidae	*Actitis hypoleucos*		3	1	14	18
Charadriiformes	Scolopacidae	*Actitis macularius*				3	3
Charadriiformes	Scolopacidae	*Arenaria interpres*			7	10067	10074
Charadriiformes	Scolopacidae	*Calidris alba*		5	370	18481	18856
Charadriiformes	Scolopacidae	*Calidris alpina*				557	557
Charadriiformes	Scolopacidae	*Calidris bairdii*				15	15
Charadriiformes	Scolopacidae	*Calidris canutus*				902	902
Charadriiformes	Scolopacidae	*Calidris ferruginea*				671	671
Charadriiformes	Scolopacidae	*Calidris fuscicollis*				381	381
Charadriiformes	Scolopacidae	*Calidris mauri*				2	2
Charadriiformes	Scolopacidae	*Calidris melanotos*				98	98
Charadriiformes	Scolopacidae	*Calidris minuta*				355	355
Charadriiformes	Scolopacidae	*Calidris minutilla*				46	46
Charadriiformes	Scolopacidae	*Calidris pusilla*		1		556	557
Charadriiformes	Scolopacidae	*Calidris temminckii*				4	4
Charadriiformes	Scolopacidae	*Gallinago delicata*				2	2
Charadriiformes	Scolopacidae	*Gallinago gallinago gallinago*	b / n	1		22	23
Charadriiformes	Scolopacidae	*Limnodromus griseus*				101	101
Charadriiformes	Scolopacidae	*Limnodromus scolopaceus*				3	3
Charadriiformes	Scolopacidae	*Limosa lapponica*				197	197
Charadriiformes	Scolopacidae	*Limosa limosa*			1	2761	2762
Charadriiformes	Scolopacidae	*Numenius phaeopus hudsonicus*				6	6
Charadriiformes	Scolopacidae	*Numenius phaeopus phaeopus*			21	474	495
Charadriiformes	Scolopacidae	*Phalaropus fulicarius*		2		84	86
Charadriiformes	Scolopacidae	*Phalaropus lobatus*				23	23
Charadriiformes	Scolopacidae	*Philomachus pugnax*				629	629
Charadriiformes	Scolopacidae	*Tringa brevipes*				15	15
Charadriiformes	Scolopacidae	*Tringa flavipes*		2		79	81
Charadriiformes	Scolopacidae	*Tringa glareola*				3	3
Charadriiformes	Scolopacidae	*Tringa nebularia*				16	16
Charadriiformes	Scolopacidae	*Tringa totanus*		2		210	212
Charadriiformes	Scolopacidae	*Tryngites subruficollis*				2	2
Charadriiformes	Sternidae	*Chlidonias leucopterus*				1	1
Charadriiformes	Sternidae	*Sterna dougallii dougallii*	b / n		1		1
Charadriiformes	Sternidae	*Sterna hirundo hirundo*	b / n	91	376	275	742
Charadriiformes	Sternidae	*Thalasseu sandvicensis*			2		2
Ciconiiformes	Ardeidae	*Ardea cinerea*		713	8	35	756
Ciconiiformes	Ardeidae	*Ardeola ralloides*		3			3
Ciconiiformes	Ardeidae	*Bubulcus ibis*		2	3		5
Ciconiiformes	Ardeidae	*Egretta garzetta*		67	19	25	111
Ciconiiformes	Threskiornithidae	*Platalea leucorodia*				5	5
Ciconiiformes	Threskiornithidae	*Plegadis falcinellus*		9			9
Columbiformes	Columbidae	*Columba livia*	b / i	25	185	272	482
Columbiformes	Columbidae	*Columba palumbus azorica*	b / END	76	43	5	124
Columbiformes	Columbidae	*Streptopelia decaocto decaocto*	b / n	1	8	127	136
Coraciiformes	Alcedinidae	*Megaceryle alcyon*		5			5
Falconiformes	Accipitridae	*Buteo buteo rothschildi*	b / END	43	4	44	91
Falconiformes	Falconidae	*Falco tinnunculus*				2	2
Falconiformes	Pandiondidae	*Pandion haliaetus*		1			1
Galliformes	Phasianidae	*Coturnix coturnix conturbans*	b / n		10		10
Gaviiformes	Gaviidae	*Gavia immer*			5	1	6
Gruiformes	Rallidae	*Fulica atra*	b / n	5077			5077
Gruiformes	Rallidae	*Gallinula chloropus*	b / n	2930	2	5	2937
Passeriformes	Emberizidae	*Plectrophenax nivalis*				1	1
Passeriformes	Estrildidade	*Estrilda astrild*	b / i	367	18	147	532
Passeriformes	Fringillidae	*Carduelis carduelis parva*	b / i	196	13	19	228
Passeriformes	Fringillidae	*Serinus canaria canaria*	b /MAC	61	136	88	285
Passeriformes	Hirundinidae	*Hirundo rustica*				9	9
Passeriformes	Motacillidae	*Motacilla cinerea patriciae*	b / END	4	35	65	104
Passeriformes	Passaridae	*Passer domesticus domesticus*	b / i	164	61	347	572
Passeriformes	Sturnidae	*Sturnus vulgaris granti*	b / END	151	41	339	531
Passeriformes	Sylviidae	*Sylvia atricapilla gularis*	b / END	22		13	35
Passeriformes	Turdidae	*Erithacus rubecula rubecula*	b / n	1			1
Passeriformes	Turdidae	*Turdus merula azorensis*	b / END	1950	72	176	2198
Procellariiformes	Procellariidae	*Calonectris borealis*	b / n	1			1
		**Abundance**		**16663**	**11793**	**54529**	
		**Species Richness**		**55**	**40**	**85**	
